# Exploring the level of lockdown fatigue and effect of personal resilience and coping behaviours on university students during the covid-19 pandemic: a cross-sectional analysis from Iraq

**DOI:** 10.1007/s12144-022-02779-8

**Published:** 2022-02-03

**Authors:** Bassam Abdul Rasool Hassan, Ali Haider Mohammed, Abdulrasool M. Wayyes, Sinan Subhi Farhan, Omar Abdulwahid Al-Ani, Ali Blebil, Juman Dujaili

**Affiliations:** 1 Department of Pharmacy, Al Rafidain University College, Baghdad, 10001 Iraq; 2 School of Pharmacy, Monash University Malaysia, Jalan Lagoon Selatan, 47500 Bandar Sunway, Selangor Malaysia; 3 Department of Anesthesia, College of Medical Science Technology, University of Mashreq, Baghdad, 10001 Iraq

**Keywords:** Lockdown-fatigue, Covid-19, Personal resilience, Coping behaviours, Students

## Abstract

Governments worldwide have imposed lockdowns in their countries to restrict unnecessary movement and curb the spread and transmission of the Covid-19 as a mitigation measure. The education sector has also implemented rapid changes, and this has transformed the operational procedures for both students and lecturers. As the pandemic has progressed, its emotional and psychological toll is equally bearing on students, leading to lockdown fatigue. This study aimed to investigate the level of lockdown-induced fatigue and its correlation with personal resilience and coping skills among university students in Iraq. This study used quantitative methods of research using a cross-sectional study design. A questionnaire survey was distributed electronically among 819 university students in Iraq. The study used three standardised scales: the lockdown fatigue scale, brief resilience scale, and coping behaviours questionnaire for data collection. Descriptive and inferential statistical analysis were done using SPSS. Overall, students indicated a high level of lockdown fatigue with a mean score of 33.48out of 50. Fear of personal safety and the wellbeing of the family was the most fear expressed by the students. The ability to go through stressful times and unpleasant events was the most common worry among the students. Female, urbanised, and science field students were the most students who suffered from lockdown-induced fatigue. However, positive coping behaviours and personal resilience were significantly correlated with decreasing fatigue levels during the lockdown period. Level of lockdown fatigue accelerated in an alarming stage among university students in Iraq. Hence, students need to build their emotional resilience and learn how to navigate surviving hard times and bounce back after a loss. This could be facilitated by counselling services being availed to educational and social institutions to benefit university students.

## Introduction

The Covid-19 pandemic poses a global health emergency, and it has rendered significant economic and health problems. Many governments worldwide have imposed stringent measures, which include mandatory lockdown, social distancing and home confinement orders to mitigate disease control and reduce the rate of transmission (Xiao & Torok, 2020). For example, starting from 1 March 2020, the Iraqi government ushered in its strategy of partial and total lockdown measures to curb the spread of Covid-19. The lockdown measures have stringent clauses, including the restriction of interprovince travel and the closure of schools and universities (Jebril, 2020). While the lockdown measures were effective in curbing the rate of transmission and the spread of the virus, they were detrimental to the mental and psychological wellbeing of people, especially young people (Marroquín et al., 2020; Volkan & Volkan, 2020). Among the varied effects of the lockdown or home confinement induced by the Covid-19 pandemic, fatigue was one of the most commonly reported effects; which was aptly described as a mental or physical state of exhaustion and lower energy levels (Labrague & Ballad, 2020; Labrague, 2021).

Lockdown fatigue is described as the impact on an individual dealing with uncertainty, anxiety, emotions, feelings, and the lack of control over the future due to the Covid-19 pandemic and imposed lockdowns (Labrague & Ballad, 2020). While the lockdown fatigue may manifest in different forms, it stems from a common cause, which is the efforts that individuals are making to acclimatise and adapt to the evolving situation of the global pandemic. The Covid-19 pandemic has affected all facets of life from its onset. People have had to change their operational procedures to comply with the regulations set by the government to contain the virus, such as lockdown and closure of non-essential facilities. This has cast a shadow of uncertainty on how the future will be like for everyone if the pandemic ends (Goldstein et al., 2021).

The education sector is not exempt from the immense regulations which the government of Iraq imposed to contain the transmission and spread of the virus. Due to the nationwide lockdown, students have had to stay home and adopt online learning (Mahase, 2020). Another non-essential activity is also restricted, limiting the alternative activities that students can participate in to pass the time. Social gatherings are prohibited, and inter-city travel is strictly monitored, leaving the majority of the students in isolation with their families. The lack of social contact has resulted in immense feelings of isolation, leading to people who have never had psychological problems developing them due to the pandemic (Aboagye et al., 2021). Furthermore, the limited amount of any alternative activity has exposed students to the full extent of the virus as they keep up with the news bulletin, witness the loss of lives and economic difficulty (Maatuk et al., 2021).

The sense of impending doom has led to psychological stress and emotional difficulties, and it is against this background, students are experiencing lockdown fatigue. Due to constant news of the loss of life and witnessing the loss of family members, the pandemic has inevitably exposed students to trauma (Field et al., 2021). However, the trauma response of individuals often varies, with other individuals quickly recovering from stressful events while others have a longer time to recover. Such challenging times would require one to have the flexibility and emotional balance, but this is not the case for most of the population as the situation constantly evolves, presenting new challenges and dynamics to live with (Abbasi, 2020). Individuals also vary in their coping behaviours; some would resort to spirituality and holistic lifestyle practices, and others would succumb to negative coping behaviours such as alcoholism and drug abuse (Pigaiani et al., 2020). Therefore, the level of lockdown fatigue among university students are still unclear, and the approaches and strategies that students may do to overcome this matter are still unidentified clearly nationally and internationally. Hence, the current study will fill this gap of knowledge by determining the lockdown fatigue and provide additional insight into the role of coping behaviour and personal resilience in facing such a challenge among university students in Iraq.

## Methods

### Study design, samples, and setting

A cross-sectional study utilising an online data collection approach was conducted during the five months of the mandatory lockdown implemented in Iraq due to the coronavirus pandemic from December 2020 to April 2021. This study included students who enrolled in colleges and universities from different provinces in Iraq. Using the Qualtrics sample size calculator, an estimation of the required sample size was performed. A sample size of at least 385 was found to be required for three predictors to attain an 80% power, with an effect size of 0.05 and alpha set at 0.05. One thousand students were initially invited from 15 universities located in different regions in Iraq to generalise the findings; however, 819 responded to our online survey (response rate = 81.9%). To qualify for the study, students had to: a) be currently enrolled in a college or university, b) be a full-time student, and c) consent to participate in the study.

### Instrument tool

Three different questionnaires using five standardised scales were used to gather data, including the lockdown fatigue scale (Labrague & Ballad, 2020), Brief Resilience Scale (Smith et al., 2008), and coping behaviours questionnaire (Savitsky et al., 2020).

#### Lockdown fatigue scale

This scale was used to evaluate signs of exhaustion associated with the lockdown or home confinement measures to slow the spread of coronavirus. The 10-item scale was answered by the participants on a 5-point Likert-type scale that ranged from 1 (never) to 5 (always). The highest possible score was 50, and the scores were categorised as indicating mild (score 1-15), moderate (score 16-30), high (score 31–45), and severe (score 46–50) fatigue. In addition, the reliability and validity of the scale were checked, where the scale’s content validity was 0.91 and the test-retest reliability was 0.93.

#### Brief resilience scale

This six-item scale determined students’ ability to bounce back from traumatic or unpleasant events associated with the pandemic and the imposed lockdown measure. Students answered the scale by responding to a 5-point Likert-type scale ranging from 0 (strongly agree) to 5 (strongly disagree). The reliability and validity of the scale were checked, where the scale’s content validity was 0.89 and the test-retest reliability was 0.90.

#### Coping behaviours questionnaire

This scale examined the ways college and university students coped during the mandatory lockdown period. The scale comprised eight items that were categorised into four dimensions: seeking information and consultation, use of humour, mental disengagement and spirituality/sources of support. Participants answered the items using a 5-point Likert-type scale that ranged from 1 (strongly disagree) to 5 (strongly agree). The reliability and validity of the scale were checked, where the scale’s content validity was 0.94 and the test-retest reliability was 0.88.

### Data collection

The Institutional Research Ethics Committee of Al Rafidain University College granted the ethical approval for this study (EC-68-2021). Since the schools were closed and teaching was online during the data collection period, an online survey was created using SurveyMonkey and sent to the students’ email addresses. Basic information about the study and the letter seeking their consent was contained on the introductory page of the online form. Names were not requested during submission to ensure the anonymity of the participants. The online survey was conducted for a period of five-month from December to April 2021, which corresponds to the six months of the mandatory lockdown measure in Iraq. Follow-up emails were sent to students on a weekly basis to remind them to complete the survey.

### Data analysis

Data completeness was checked before entering into SPSS version 26. To quantify the data, we calculated frequencies, standard deviations, and weighted means. The normality of the data distribution was confirmed via the Kolmogorov-Smirnov test. Bivariate analysis was facilitated using the independent t-test, and Pearson’s correlation coefficient (r) to examine correlations between key study variables. The level of statistical significance was set as *P* < 0.05.

## Results

### Socio-demographic characteristics

Students from universities and tertiary institutions around Iraq were approached to participate in this survey. Just over half of the respondents were male (50.4%), and nearly all respondents were of Arabic ethnicity (95.4%). The majority of the students were living in urban areas (91.3%), and the most dominant field of study was STEM (80.4%). The mean age was 23.65 ± 6.31, and the means scores were just over 3 for lockdown fatigue, personal resilience and coping skills (Table 1).

**Table 1:** Socio-demographic characteristics of respondents (*n* = 819)

Characteristic		Frequency (%)
Gender	Male	413 (50.4)
	Female	406 (49.6)
Ethnicity	Arab	781 (95.4)
	Kurdish	38 (4.6)
Type of university/college	Public	146 (17.8)
	Private	673(82.2)
Type of residence	Urban	748 (91.3)
	Rural	71 (8.7)
Study field	STEM (e.g. Science, Technology, Engineering and Mathematics)	659(80.4)
	Non-STEM(e.g.Humanities, Arts, Literature and Management)	160 (19.6)
	Mean	SD
Age	23.65	6.31
Lockdown fatigue score	3.34	0.67
Personnel resilience score	3.36	0.58
Coping skills score	3.47	0.59

### Participants’ lockdown fatigue

The participants were asked how they feel during the lockdown period induced by the Covid-19 pandemic. One of the most dominant feelings which the students experienced is the fear of their own family’s safety and wellbeing (mean = 3.87). The students also indicated that facing feelings of sadness and depression and fearing that “this pandemic will never end soon “(mean = 3.5; 3.4) are the other most indicators that exhausted them during the lockdown period. On the other hand, the students reported that the feelings which they had the least were loss of sleep over the pandemic and any headaches or body pains (mean = 2.8; 3.0). The students also felt having a certain level of difficulty in concentrating or being distracted easily amid lockdown periods. Overall, based on the scale, the total lockdown means is 33.48, indicating that students have a high level of lockdown fatigue (Table 2).

**Table 2:** Responses of participants on the Lockdown Fatigue Scale (n = 819)

Items	Minimum	Maximum	Mean	Std. Deviation
I worry a lot about my personal and family’s safety during this pandemic	1.00	5.00	3.8657	1.15605
I have difficulty concentrating and distracted easily	1.00	5.00	3.3162	1.08281
I frequently felt weak or tired as a result of this lockdown	1.00	5.00	3.3736	1.08764
I have been feeling irritable	1.00	5.00	3.3187	1.17108
I have difficulty falling or staying asleep over thinking about this pandemic	1.00	5.00	2.8144	1.26539
I have felt sad and depressed as a result of this lockdown	1.00	5.00	3.5079	1.17570
I have been losing my interests to do the usual things I love	1.00	5.00	3.3578	1.22033
I have been experiencing a general sense of emptiness	1.00	5.00	3.3919	1.09744
I have been experiencing headaches and body pains	1.00	5.00	3.0855	1.21322
I have thoughts that this pandemic will never end soon	1.00	5.00	3.4530	1.22771
Total lockdown fatigue score	10	50	33.48	6.60

### Participants’ personnel resilience

The participants were asked how they bounce back from traumatic or unpleasant events associated with the pandemic and imposed lockdowns (Table 3). Participants indicated that their greatest difficulty was coping through the duration of stressful and challenging times imposed by the pandemic. The highest mean score was recorded on the item “I have a hard time making it through stressful events” (mean = 3.44). This was followed by the ability to snap back after something terrible happens to the students. The participants, however, indicated that as the hard times subside, they are better at bouncing back into wellbeing. The least mean score was recorded on the item “I tend to bounce back quickly after hard times” (mean = 3.21).

**Table 3:** Responses of participants on the Brief Resilience Scale (*n* = 819)

Items	Minimum	Maximum	Mean	Std. Deviation
I tend to bounce back quickly after hard times	1.00	5.00	3.2198	1.11800
I have a hard time making it through stressful events	1.00	5.00	3.4432	1.06518
It does not take me long to recover from a stressful event	1.00	5.00	3.2991	1.10329
It is hard for me to snap back when something bad happens	1.00	5.00	3.4212	1.10990
I usually come through difficult times with little trouble	1.00	5.00	3.3907	1.04248
I tend to take a long time to get over setbacks in my life	1.00	5.00	3.3944	1.19984

### Participants’ coping skills

The participants were asked which of the activities they resorted to in coping with the mandatory lockdown period. “I put my trust in God” was the most common action that participants did to cope during the lockdown (mean = 4.33). This was followed by searching for information on any questions which they had pertaining to the lockdown. The participants also resorted to finding alternative activities to distract them from the events occurring and seeking emotional support from friends and relatives. The least common action was resorting to alcohol and drugs to feel better (Table 4).

**Table 4:** Participants’ responses on the Coping Behaviours skills (*n* = 819)

Items	Minimum	Maximum	Mean	Std. Deviation
I try to get advice from someone about what to do	1.00	5.00	3.4853	1.12300
When I have a question about the situation, I search for information	1.00	5.00	3.7914	1.08062
I use alcohol or drugs to make myself feel better	1.00	5.00	2.3444	1.45595
I put my trust in God	1.00	5.00	4.3346	1.07077
I turn to work or other substitute activities to take my mind off things	1.00	5.00	3.5702	1.06334
I eat more than usual to calm myself down	1.00	5.00	3.2996	1.20810
I enjoy the jokes about the situation	1.00	5.00	3.4284	1.08694
I try to get emotional support from friends or relatives	1.00	5.00	3.5361	1.17427

### Correlation between lockdown fatigue and key study variables

The difference and correlation between lockdown fatigue and key variables among the participants were analysed (Table 5). Among the variables tested, the mean differences among gender, ethnicity, type of residence and study field and lockdown fatigue are significantly different. We conclude that female, urbanised, and science field students had a higher fatigue level than their reference. In contrast, the Pearson r correlation test showed that there is a significant (*P* < 0.05), positive moderate and strong correlation (*r* = 0.52; 0.74) between lockdown fatigue and personnel resilience and coping skills, respectively.

**Table 5:** Difference and correlation between lockdown fatigue with other scales and demographic variables

Characteristics	Categories	Mean (SD)	Test Statistics	*P* value
Gender^&^	Male Female	32.71 (6.24) 34.27 (6.87)	−3.42	0.001*
Type of university^&^	Public Private	32.94 (5.92) 33.60 (6.73)	−1.10	0.272
Type of residence^&^	Urban Rural	33.73 (6.55) 30.96 (6.67)	3.42	0.001*
Study field^&^	STEM Non-STEM	35.45 (6.58) 33.63 (6.68)	−3.25	0.001*
Personnel resilience score mean^#^	–	–	0.52	0.0001*
coping skills score mean^#^	–	–	0.74	0.0001*

&t-test for independent group

#Pearson *r* correlation

*Significant value (*p* > 0.05)

## Discussion

Lockdown fatigue among individuals is a growing concern for the healthcare community as the pandemic persists with new variants of the coronavirus emerging. Despite evidence that young people are more likely to develop fatigue due to lockdown measures, there are no studies on how individual resilience and coping skills reduce fatigue in university students conducted in the Middle East, particularly in Iraq. Lockdown fatigue is a psychological phenomenon that has stemmed from the Covid-19 pandemic and increasingly affects students (Labrague, 2021). With the pandemic coming in waves and an upsurge of new infections, it would likely lead to lockdowns in the future for an uncertain period. One of the significant concerns, that Iraqi students have worried about during the lockdown period, is the fear of personal and family safety. Students were often concerned with whether they will survive the pandemic without contracting the virus (Amanzio et al., 2021). They would also be worried whenever they experience symptoms similar to those of the Covid-19 virus, such as headaches, sore or dry throat, fevers and low energy levels. This concern is heightened in students with family members who are at a higher risk of contracting the virus. These family members with diabetes, high blood pressure, cancer and other immunosuppressive conditions have expressed their distress and essential workers in the healthcare industry (Majumdar et al., 2020; Mohammed et al., 2020). Additional stress comes from having to cope with individuals who need to undergo treatment from the virus, given that they would need to be cautious due to counteractions of medication. Thus, chances of recovery would be compromised. Internationally, there is still an immense concern over individuals who have been working through the pandemic as essential workers (Vishwakarma et al., 2021). Their families would have anxiety over their safety from contracting the virus and recovery from illness. This is consistent with the study of Pachiyappan et al., which highlighted that students who have family members who are essential workers in the healthcare settings were found to be more concerned over the virus. This anxiety would heighten in periods where there are high death tolls and surges in new infections (Pachiyappan et al., 2021).

Students demonstrated feelings of sadness and depression due to the lockdown, and this is rationalised by the complex set of circumstances that they are facing. The education system has been disrupted, and there have been measures such as virtual learning, which have been introduced to ensure continuity in the education system. However, virtual learning is not as convenient as it is presumed to be (Sharma et al., 2020). While it may be considered simply stopping face-to-face classes and switching to learning from home, it requires much more commitment and has its own challenges. Similar notions are observed from the findings of Bu et al., in the United Kingdom, and Reicher and Drury, which explored how studying from home during the pandemic would be for students under the stress of the pandemic (Bu et al., 2020; Reicher & Drury, 2021). For freshmen, not all students live in stable homes which are conducive for studying. Family dynamics and living arrangements often curb the efficacy of virtual learning. In addition, learning from home while also facing economic difficulties, challenges and illnesses limit the engagement of students. This has resulted in students struggling to keep up with their performance at school and even losing interest in studying (Naddeo et al., 2021).

Moreover, our findings highlighted that female student had a high level of fatigue compared to the male student. This is probably because of gender differences in the expression of sentiments and emotions such as concern, fear, sadness, anxiety, and pain and bodily discomfort. Men, according to several pieces of evidence, tend to suppress their emotions and feelings, whereas women are more vocal in expressing their emotions (Chaplin et al., 2008; Tolin & Foa, 2008). This finding confirms a long-held gender stereotype in Iraqi culture, according to which the expression of thoughts, feelings, and emotions is more acceptable for women than for men. Therefore, this study highlights the critical need to implement gender-tailored strategies to successfully treat the adverse impacts of the lockdown measure and reduce fatigue. Besides that, other groups of students who significantly reported a high level of fatigue are science students and those who are residents in urban areas. Empirical studies had proved that major scientific students are more stressed and exhausted than non-science students. This is because they have a high workload, more lessons and labs, respecting deadlines, balancing university and more private life (Portoghese et al., 2019). These pressures have been related to a higher risk of depression and lower academic performance, consequently worsening their level of fatigue during the lockdown period.

Another important finding was the positive moderate correlation between university students’ personal resilience skills and lockdown fatigue. Personal resilience is essential as it enables one to navigate challenges and unpleasant circumstances to continue accomplishing their goals. Our students’ sample demonstrated that they have a hard time making it through stressful events. Hence, the personal resilience of students has been challenged by the lockdown, as they have to continue with a common journey, which is their education. This is tested by how the students have continued studying and their rate of success (Labrague et al., 2020). Overall, students have been able to continue with their improvised methods of study, improving students’ ability to self-study and research. However, the mental toll and pressure of continuing with education despite personal hardships and failure have been a key feature of the pandemic (Savitsky et al., 2021). Individuals’ time to recover from setbacks varies, which differentiates the students who are able to cope and those who are still unwell from the lockdown period (Cosmas, 2020). A previous scholar conducted by Migliorini et al. indicated how resilience had been tested in students, especially those on the brink of completing their studies who have an uncertain future to navigate (Migliorini et al., 2021). Therefore, the lockdown fatigue dampens the motivation of students to study or be proactive in their studies.

Individuals often develop both conscious and non-conscious coping mechanisms when they are going through unpleasant and stressful events. This was supported in our study, where coping skills was strongly correlated with the level of lockdown fatigue. This probably occurs due to the reality that some situations cannot be changed or be easily remedied; therefore, the individual is the one who has to adjust and cope with the stressful situations (Zhang et al., 2021). Ideally, coping mechanisms should be positive in helping the individual cope without inflicting personal harm in both the short and long term. Negative coping mechanisms such as binge-eating, alcohol and drug abuse are temporarily numb the individual from the current situation, but shortly after they inflict harm on the individual rather than helping them (Khan, 2021). From our present study, one of the most common coping mechanisms adopted by the students was placing their trust in God, which is, resorting to spirituality to search for answers for this dire situation. While the concept of divinity is varied among individuals, the commonly held belief is that resorting to divinity in stressful times would lead to relief, guidance and protection (Kadir, 2020). This is consistent with the study of Molteni et al., which indicates that in the period surrounding the lockdown, people of different faiths have been increasingly committed to their various religions as they seek relief from the pandemic (Molteni et al., 2021). Students who practised their religion regularly were more resilient than those who did not have any strong ties to any religion (Prazeres et al., 2021).

Apart from religious activities, students have also been occupying their minds with various activities during the stressful situations in lockdown. Notably, students have been using social media and participating in various activities which build a sense of community to curb the loneliness introduced by the lockdown (Nitschke et al., 2021). The discovery of new hobbies and indoor activities has been a key factor in coping with the mental fatigue induced by the lengthy lockdown. Studies by Devi indicate that due to the lockdown and restrictions, individuals often develop a timetable that enables them to spend their day without amplifying the abnormality such as waking up later, being slower in their routines and increased interest in home-care and wellbeing (Devi, 2020). These activities make it feasible for individuals to find emotional support from friends and relatives through socialising. However, this would be dependent on the emotional capacity of the friends and relatives, given that the pandemic and lockdown has affected all facets of society in drastic ways (Amanzio et al., 2021).

Lastly, another interesting coping approach that students were doing during the lockdown period is searching for information to gain insight into how they can effectively deal with the pandemic. Popular searches include ‘number of Covid-19 cases’ and ‘home remedies’ (Chandra, 2020). Students have also been lately keen on learning about the various vaccinations which are available and how they would work. With vast information available on the internet, students also enjoy sharing their knowledge with companions leading to lengthy conversations about the pandemic. This would give the individual seeking information a sense of control over the situation as they would not be susceptible to any factors which they are unaware of (Wasil et al., 2021).

### Strength and limitations of the study

The survey was distributed across 15 universities located in the south, north, east, and west of Iraq so that findings can be generalised as it was representative of the university and college students in Iraq. The limitations of this study are the evolving scope of the Covid-19 pandemic and the possibility of future lockdowns occurring. The uncertainty of the period in which the pandemic will exist is also another limitation of the study. In addition, the responses gathered from this study are based on emotional responses; thus, they may be prone to bias; however, this is addressed by choosing a sufficient sample size for the study.

## Conclusion

The imposition of compulsory lockdown and home confinement as a strategy to reduce the rate of Covid-19 transmission led university students to experience significant levels of fatigue in Iraq. This study provided tangible evidence which links higher personal resilience and coping skills among university students with lower levels of fatigue triggered by the lockdown. From the results of this study, it is necessary to instigate activities and methods which build personal resilience and foster positive coping strategies as methods of reducing high levels of lockdown fatigue and all detrimental mental and psychological issues related to the pandemic. Furthermore, government authorities and regulators should continuously evaluate the efficacy of the measures stipulated by the lockdown and strategies on how these measures can be eased without endangering students’ health.

## Data Availability

Data and other materials are available upon request from the corresponding author.
